# A brief history of ependymoma

**DOI:** 10.1093/neuonc/noag016

**Published:** 2026-01-31

**Authors:** David R Ghasemi, Denise Obrecht-Sturm, Kimberly M Wallgren, Martin U Schuhmann, Beate Timmermann, Stefan Rutkowski, Ulrich Schüller, Kristian W Pajtler

**Affiliations:** Department of Pediatric Hematology and Oncology, University Medical Center Hamburg-Eppendorf, Hamburg, Germany; Research Institute Children’s Cancer Center Hamburg, Hamburg, Germany; Mildred Scheel ­Cancer Career Center HaTriCS4, University Medical Center Hamburg-Eppendorf, Hamburg, Germany; Hopp Children’s Cancer Center Heidelberg (KiTZ), Heidelberg, Germany; Department of Pediatric Hematology and Oncology, University Medical Center Hamburg-Eppendorf, Hamburg, Germany; Collaborative Ependymoma Research Network (CERN), A Program of the National Brain Tumor Society, Newton, Massachusetts, USA (K.M.W.); Section of Pediatric Neurosurgery, Department of Neurosurgery, University Hospital Tuebingen, Tuebingen, ­Germany (M.U.S.); Department of Particle Therapy, University Hospital Essen, West German Proton Therapy Centre Essen (WPE), West German Cancer Center (WTZ), Essen, Germany; German Cancer Consortium (DKTK), Heidelberg, Germany; Department of Pediatric Hematology and Oncology, University Medical Center Hamburg-Eppendorf, Hamburg, Germany; Department of Pediatric Hematology and Oncology, University Medical Center Hamburg-Eppendorf, Hamburg, Germany; Research Institute Children’s Cancer Center Hamburg, Hamburg, Germany; Institute of Neuropathology, University Medical Center Hamburg-Eppendorf, Hamburg, Germany; Hopp Children’s Cancer Center Heidelberg (KiTZ), Heidelberg, Germany; Division of Pediatric Neurooncology, German Cancer Research Center (DKFZ) and German Cancer Consortium (DKTK), Heidelberg, Germany; Department of Pediatric Oncology, Hematology, Immunology, and Pulmonology, Heidelberg University Hospital, Heidelberg, Germany; National Center for Tumor Diseases (NCT) Heidelberg, A Partnership Between DKFZ and Heidelberg University Hospital, Heidelberg, Germany

**Keywords:** ependymal tumors, ependymoma, medical history, molecular classification

## Abstract

Ependymoma represents a biologically and clinically heterogeneous group of glial tumors that arise throughout the whole neuroaxis and in all age groups. Whereas intracranial ependymoma is usually found in children, adults suffer mostly from spinal cord ependymoma. In comparison to other neuro-oncological tumor entities, ependymoma has been largely understudied for decades. However, the recent years resulted in unprecedented progress with regard to the understanding of the biological underpinnings and clinical features of ependymoma. Here, we review the history of ependymoma research with a focus on the development of classification models throughout the years and a discussion of the most important clinical trials that have led to the current therapeutic standard, comprising maximal safe resection and, in most cases, radiotherapy. Critically, the evidence for effective drugs and chemotherapies in ependymoma is still sparse. However, these important questions may be soon addressed with the finalization of the currently unpublished last generation of multi-institutional trials in Europe (SIOP EP II) and Northern America (ACNS0831). Lastly, we discuss the current challenges in the field of ependymoma research and the necessary next steps toward the goal of findings cures for all types of ependymal tumors.

Key Points

- Historically, ependymoma research largely consisted of refining disease classification.
- The understanding of the biology of ependymoma has markedly improved in recent years.
- Molecular classification offers new possibilities to design future ependymoma trials.

Ependymoma comprises a heterogenous group of glial tumors that accounts for 3% of all primary malignancies of the central nervous system (CNS).^1^ Although rare in absolute numbers, ependymoma represents the third most common malignant brain and the most common spinal cord tumor in children as well as the third most common primary spinal cord tumor in adults.^1^ In comparison to other malignant CNS tumors, ependymoma has not received the same attention by the research community and remains understudied. This is exemplified by the number of scientific publications on different tumor entities. Whereas 30 281 publications in the Pubmed database contained the term “glioblastoma” and 5114 the term “medulloblastoma” in their titles, only 2312 studies were found under the term “ependymoma” (29/07/2025).

Throughout the last years, the understanding of the ­biological underpinnings of ependymoma has grown substantially and culminated in a molecular classification system that by now includes at least 10 groups.^2–4^ For some of these, characteristic molecular alterations have been identified, such as *ZFTA-* and *YAP1-*associated oncogenic fusions in supratentorial ependymoma (ST-ZFTA/ST-YAP1), over­expression, or rarely, mutations of *EZHIP* or *H3K27M* in Posterior Fossa Group A ependymoma (PF-A), and mutations in *NF2* as well as amplifications of *MYCN* in groups of spinal ependymoma (SP-MYCN).^2^^,^^3^^,^^5–7^ The prognosis of ependymoma differs strongly based on molecular and clinical risk factors. Due to the rarity of the disease and the retrospective nature of many studies that investigated its clinical course, it remains difficult to define exact survival rates. For intracranial ependymoma, 10-year overall survival (OS) rates as low as 52% have been reported.^8^ Apart from the highly aggressive entity SP-MYCN, spinal ependymoma generally shows a more favorable long-term prognosis than intracranial ependymoma but can result in high morbidity, especially if relapses occur. Generally, ependymoma is prone to very late relapses that can arise more than 10 years after the end of primary treatment.^9^^,^^10^ The current gold standard for ependymoma therapy remains maximal safe resection in combination with adjuvant radiotherapy for the ­majority of patients.^8^ The efficacy of chemotherapy is disputed, as ependymoma is generally considered a chemotherapy-resistant tumor and different regimens have shown only modest success in clinical trials.^11–15^ No targeted treatment approaches have been established for ependymoma. In the case of relapse, therapeutic options are limited, and survivors frequently suffer from lifelong disease- and therapy-related sequelae.^16^^,^^17^ Thus, there is a substantial clinical need to develop better risk stratification and new risk-adapted therapies for these patients.

In this review, we aim to present the history of research on ependymoma, to summarize the progress that has been made along the way, and to discuss the challenges that must be overcome to arrive at a cure for all ependymoma patients while limiting treatment sequelae. We focus on the historical development of the ependymoma classification and treatment, as the details of the molecular underpinnings of the different ependymoma groups have been extensively reviewed elsewhere.^8^

## The Roots of Ependymoma Research

Traditionally, the name “ependymoma” has been given to tumors that showed morphological similarities to ependymal cells of the ventricle system, which were recognized as a distinct cell type in the 19th century.^18^ In 1865, the German pathologist Rudolf Virchow, who also coined the term “glioma,” suggested that ependymal tumors may be congenital or caused by chronic irritation and trauma to the ependyma.^19^ He also described a case of an infratentorial ependymoma that arose from the ependyma of the fourth ventricle and resulted in hydrocephalus. Prior to Virchow, scientific reports on ependymoma were mostly constricted to single case studies. It took until the beginning of the 20th century for ependymoma research to start gaining traction. In 1899, the first description of perivascular rosettes and pseudorosettes and so called “blepharoblasts” was published.^20^ As of today, true ependymal rosettes and perivascular pseudo-rosettes remain as two of the most characteristic histopathological features of ependymoma. Around the same time, multiple researchers suggested a connection between glial tumors and ependymal precursor cells.^21^^,^^22^ At this point, the existence of a distinct type of ependymal CNS tumor seemed to be widely accepted within the field of neuro-oncology, which was still in its infancy.^23^^,^^24^

## Early Debates, First Classification Attempts, and New Subgroups

A first systematic overview of ependymal tumors was ­presented by Percival Bailey and Harvey Cushing as part of their ground-breaking classification of CNS tumors.^25^ Interestingly, Bailey emphasized the diagnostic challenges concerning ependymoma as early as 1924 in a detailed description of the histopathological characteristics of ependymal tumors and doubted that many of the published case reports were correctly diagnosed.^26^ In opposition to other authors, who had argued that ependymoma with malignant properties and diffuse growth pattern existed, Bailey and Cushing agreed with Virchow in regarding ependymoma as a generally benign tumor.^24–27^. However, for the first time they differentiated between ependymoma and a clinically more aggressive embryonal ependymal tumor, which they called “ependymoblastoma.”^25^ The existence of a distinct ependymoma variant of embryonal origin would remain disputed for decades to come, until it was eventually resolved by introducing the new entity “embryonal tumor with multi-layered rosettes” (ETMR) in 2016.^28–32^ In 1927, Bailey revised his proposed division into ependymoma and ependymoblastoma and recommended to not use the term “ependymoblastoma” anymore, before in 1938 Kernohan reintroduced it in the context of the publication of a large case series.^27^^,^^ 33^

Throughout the first half of the 20th century, a lively debate concerning the optimal classification of ependymoma remained ongoing. For instance, the French physicians Roussy and Oberling proposed an alternative classification system, in which ependymomas were grouped together with choroid plexus carcinoma and subsequently divided into the three subgroups “ependymocytoma,” “ependymomaglioma,” and “ependymoblastoma.”^34^^,^^35^ In 1932, Kernohan described for the first time myxopapillary ependymoma of the spinal cord, and in 1945, Scheinker identified subependymomas as a distinct variant from other ependymal tumors.^36^^,^^37^ Both entities are still used until today. Early attempts to create a classification system based on malignancy grading criteria instead of histological ­features were largely abandoned.^38^

In the 1950s, Bailey and Cushing’s groundbreaking work was continued by Klaus Joachim Zülch, who had also worked on ependymoma in adolescents before.^39^ Based on a new and expanded cohort of CNS-tumors, he proposed a revised and improved classification system that should become the basis for the first WHO classification and also included a chapter on ependymoma.^40^ Zülch highlighted the clinical heterogeneity within the spectrum of ependymal tumors and suggested that supratentorial ependymoma may represent an aggressive subtype. However, he also felt that, at this point, the classification of ependymoma was largely complete—culminating in a statement that concerning ependymomas, a further subdivision would be neither morphologically nor biologically necessary.^40^

## Ependymoma as a Changing Entity in the WHO Classifications Between 1979 and 2007

**Figure 1.**
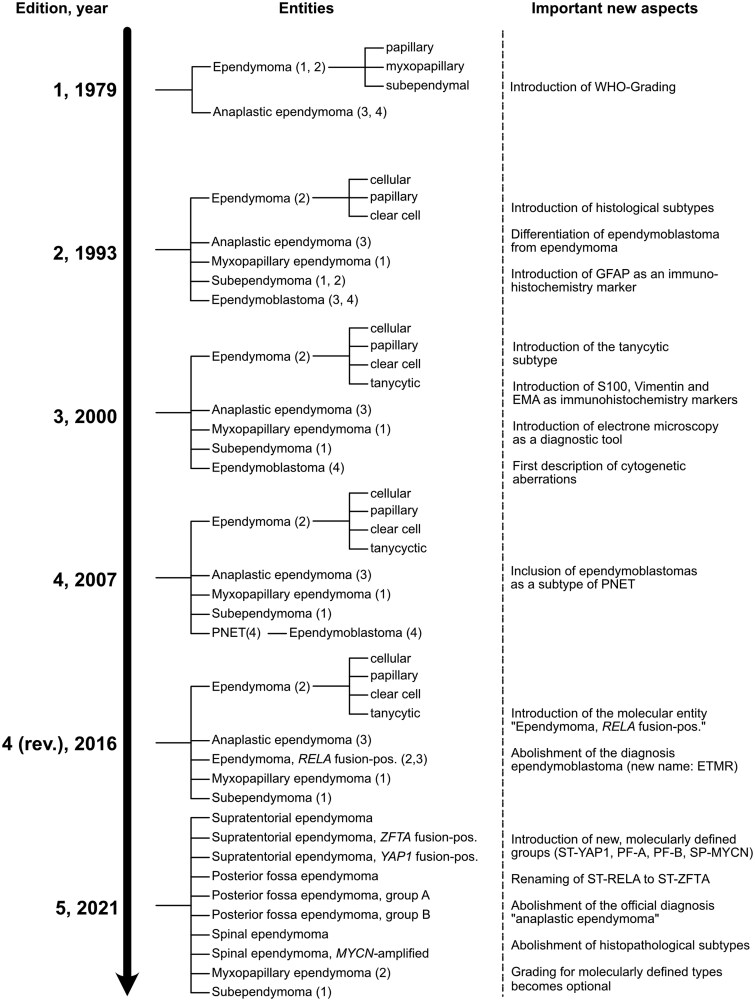
A graphical summary of the development of the classification of ependymal tumours throughout the WHO classifications from 1979 to 2021.

Throughout the following years, it became evident that an international harmonization of the classification of CNS tumors was necessary to make sure that studies were comparable and histopathological stratification reproducible across different countries and centers. The publication of the first version of the WHO classification of CNS tumors in 1979 represented a significant step toward harmonizing the previously very heterogeneous approaches of classifying brain and spinal cord tumors (Figure 1).^41^ Regarding ependymoma, one of the major differences to the previous classification by Zülch was the introduction of a grading system: Now, two variants were distinguished from another: On the one hand, ependymoma with WHO grades 1 and 2, which was further stratified into myxopapillary, papillary and subependymal subtypes, and on the other hand, anaplastic ependymoma, whose corresponding WHO grade was 3 or 4. The characteristic ependymal rosettes and perivascular pseudo-rosettes remained as an important diagnostic criterion. Despite introducing grading for ependymoma, the authors emphasized the potential pitfalls of using it for ­clinical decision-making and prognostication.^41^

**Figure 2.**
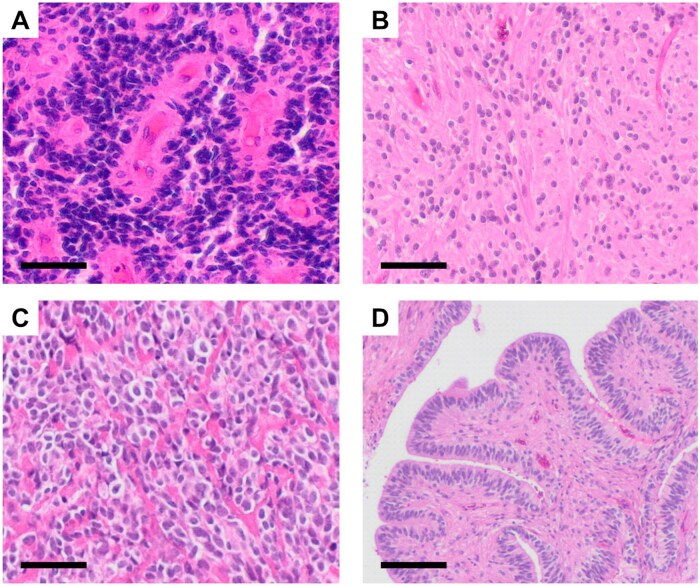
The historic histopathological spectrum of ependymoma. Representative scans of hematoxylin and eosin stainings of (A) anaplastic, (B) tanycytic, (C) clear cell, and (D) papillary ependymoma. Scale bars in A-C correspond to 50 µm, and scale bar in D corresponds to 100 µm.

For the following 3 decades, a classification of epen­dymoma based on histopathological features and grading remained the gold standard. Under the leadership of Paul Kleihues, the following 2 versions of the WHO classification in 1993 and 2000 introduced new subtypes with the variants of clear cell, cellular, and tanycytic ependymoma (Figure 2), differentiated anaplastic ependymoma from ependymoblastoma, which was reintroduced as an independent entity mainly based on the work of Lucien Rubinstein, and made changes to the grading criteria (Figure 1).^29^^,^^30^^,^^42–44^ Whereas the second edition only described the immunoreactivity patterns for GFAP, the third edition included information on a broader panel of immunohistochemistry markers, namely GFAP, EMA, S100, Vimentin, and Nestin. Furthermore, it was reported that ependymoma do not express neuronal antigens, such as synaptophysin. To date, GFAP, EMA, and S100 are widely used as robust expression markers. As compared to the second edition, the third edition also described ultrastructural features of ependymoma as defined by electron microscopy.^43^ However, neither immunohistochemical nor ultrastructural markers were influencing the overall stratification system, which remained entirely based on the histopathologic appearance alone.

The third edition of the WHO classification in 2000 was the first one to summarize genetic aberrations in ependymoma, as a correlation between the NF2-syndrome, aberrations on chromosome (Chr.) 22, and a higher incidence of spinal ependymoma was reported. Despite the new adaptions to ependymoma classification, the elephant in the room remained: Still, there was no clear evidence that the widely adapted grading system had prognostic significance, and there were no robust biomarkers that could be used in the clinical setting to confidentially assess an individual patient’s risk profile. This issue was underlined by an important study led by David Ellison and Richard Grundy in 2011 which showed that grading in ependymoma resulted in high inter-observer variability and was only of limited clinical use.^45^ However, it should be noted that the evidence base for histopathological grading in ependymoma shows mixed results. Several studies have reported prognostic relevance for grading, whereas others did not, and this will be further discussed later in this review.

## The Molecular Era, Part 1: The Development of a New Classification System

**Figure 3.**
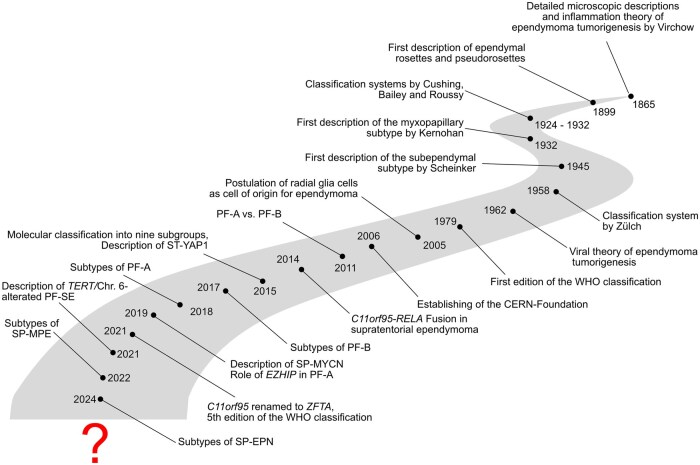
Visualization of important milestones of ependymoma classification in the context of other notable discoveries related to ­ependymal tumors.

The fourth edition of the WHO classification in 2007 did not introduce larger adaptations to the status quo with regard to ependymoma apart from placing ependymoblastomas under the umbrella term “primitive neuroectodermal tumor” (Figure 1).^46^ Notably, radial glia were identified as the most probable cell of origin for ependymoma around this time.^47^ The classification of ependymoma was about to change dramatically in the years to come. Similar to other CNS tumors, the introduction of next generation sequencing methods and particularly DNA methylation-profiling led to a rapid and profound overhaul in the way that ependymoma was classified and to a so far unprecedented understanding of its biological underpinnings (Figure 3). A first major step toward a molecularly based classification system was the description of 2 molecularly and clinically distinct types of posterior fossa ependymoma, called PF-A and PF-B.^48^ Shortly after, Parker et al. identified gene fusions between *C11orf95 (*now *ZFTA)* and *RELA* as the main drivers of supratentorial ependymoma.^49^ In 2015, a new molecular classification model was proposed that defined 9 distinct molecular groups that were spread across the 3 major compartments of the CNS. Importantly, these groups were not only biologically but also clinically relevant and showed significant prognostic differences.^2^^,^^50^ Furthermore, a new type of supratentorial ependymoma was identified: ST-YAP1, which occurs primarily in infants and harbors fusion genes that include the gene *YAP1*.^2^^,^^7^^,^^51^ Subsequent studies showed that the former histopathological subtypes partly overlapped with the new molecular groups,^52^^,^^53^ which could be robustly diagnosed by methylome and transcriptome analysis. Still, the new molecular quickly outcompeted the old histopathological classification system.

Whereas this study represented a major step forward for the field of ependymoma research and was largely accepted in subsequent publications, it was not yet fully adopted in the 4th revised edition of the WHO classification in 2016 (Figure 1).^32^^,^^54^ However, this edition incorporated several important novelties as compared with the previous version from 2007: Firstly, after almost 100 years of dispute, the diagnosis ependymoblastoma was again—and until to date finally—abandoned and replaced by the new entity ETMR, after studies had shown a lack of specificity and that the term “ependymoblastoma” was used to describe a number of different primitive CNS malignancies.^31^ Additionally, a new diagnosis was introduced: supratentorial ependymoma with *RELA-*fusion. The establishment of the first molecularly defined ependymoma diagnosis represented a paradigm shift and the next step of a switch toward a molecular-based classification system.^55^

From 2015 on, several studies further developed the ­original classification scheme. Based on DNA methylation-profiling, 2 papers sub-stratified PF-A and PF-B into subtypes,^6^^,^^56^ and a small subgroup defined by *ACVR1-*mutations was described.^57^ Importantly, overexpression of *EZHIP (Cxorf67)* and a loss of *H3 K27-*trimethylation were identified as main molecular drivers of aggressive PF-A.^5^^,^^6^^,^^58^^,^^59^ Recently, the cIMPACT-NOW group proposed a loss of H3 K27-trimethylation as an essential and EZHIP expression as a desirable marker to distinguish PF-A from PF-B.^60^ In 2019, 3 studies established a new type of spinal ependymoma with dismal prognosis that was characterized by a *MYCN-*amplification (SP-MYCN).^3^^,^^61^^,^^62^ For supratentorial ependymoma, several studies described novel fusion genes involving the gene *C11orf95/ZFTA*,^63–66^ potential new subtypes of supratentorial ependymoma^63^ and investigated the mechanisms in which *ZFTA-*associated fusions contribute to oncogenesis.^63^^,^^67^^,^^68^ In addition to the establishment and refinement of the new classification system, there was also significant progress with regard to the identification of novel biomarkers for clinical risk stratification and histopathological surrogate markers for the methylation groups: Whereas Chr. 1q-gains had been identified as an unfavorable prognostic marker as early as 2002, Chr. 6q-losses in PF-A as well as homozygous losses of *CDKN2A* in ST-ZFTA were now proposed as dismal prognostic features as well.^69–74^ In order to make the new classification broadly available, several attempts to establish surrogate markers for the methylation groups were made: A loss of H3 K27-trimethylation as identified by immunohistochemistry was shown to distinguish PF-A from PF-B.^75^ For supratentorial ependymoma, immunoreactivity for L1CAM was proposed to identify ST-ZFTA. However, later studies showed that although L1CAM is a useful marker to distinguish ST-ZFTA from ST-YAP1, it is expressed by other CNS tumors as well and is positive across ST-ZFTAs with variable fusion partners, therefore limiting its utility as an isolated biomarker.^76–79^ Unlike L1CAM, p65, another potential marker for ST-ZFTA, was shown to be negative in most cases that harbored “atypical” *ZFTA-*fusions.^63^^,^^80^ SP-MPE could be confidently identified using positive expression of HOXB13.^81^^,^^82^

The rapid progress that was made between 2010 and 2020 culminated in the 5th edition of the WHO classification in 2021, which introduced the so far most significant changes in ependymoma classification since the first version in 1979 (Figure 1).^4^^,^^83^^,^^84^ Out of 10 groups, 5 were molecularly defined (ST-ZFTA, ST-YAP1, PF-A, PF-B, SP-MYCN). ST-ZFTA, which had formerly been designated ST-RELA, was renamed after the above-mentioned studies had shown that *ZFTA (C11orf95*) was the more frequent fusion partner, whereas *RELA* could be replaced by genes such as *MAML2, MAML3, NCOA1,* or *NCOA2*. Subependymoma and myxopapillary ependymoma could still be diagnosed based on histopathological features alone and kept a grade, whereas grading became optional for molecularly defined types. DNA-methylation profiling was designated the status of gold ­standard for ependymoma classification.^85^ Since global methylome analysis was (and still is) not available everywhere and would not always result in a robust classification, the suffixes “not otherwise specified” (NOS) and “not elsewhere classified” (NEC) were introduced for labeling respective cases.^4^ The histological variants “tanycytic,” “papillary,” “clear cell,” and notably also “anaplastic” ependymoma were abandoned. In summary, these changes almost completely shifted the classification of ependymoma from a histopathologic to a molecular system.

## The Molecular Era, Part 2: Refinement and Validation

The years since 2021 have shown that further refinements of this classification system are still possible, particularly with regard to spinal ependymoma: In 2022, SP-MPE was stratified into 2 clinically relevant subtypes,^82^ and in 2024 a study showed that SP-EPN can also be sub-stratified.^86^^,^^87^ Furthermore, a limited case series described a potentially novel subtype of MYCN-positive spinal ependymoma without *MYCN-*amplification.^88^ One study used RNA-sequencing instead of DNA methylation-profiling to reveal further ­heterogeneity of supratentorial ependymoma on the transcriptomic level.^89^ For infratentorial ependymoma, a group of tumors with a subependymal or mixed phenotype, *TERT-*promotor mutations and Chr. 6 loss was described.^90^ In addition, more recent studies revealed that *ZFTA-*fused ependymomas can arise in the posterior fossa, further questioning the established narrative that biologically distinct groups of ependymal tumors are restricted to specific anatomical compartments.^60^^,^^91^^,^^92^ Furthermore, new biomarkers, such as Biglycan-positivity, have been proposed as potential additions to the current risk-stratification.^93^ Whether these new developments will eventually be incorporated into the 6th edition of the WHO classification, remains to be seen. Recently, the cIMPACT-NOW group has proposed convincing criteria for establishing new CNS tumor types, and to date it is unclear whether any further ependymal tumor groups will fulfill these.^94^ Importantly, by now most of the relevant diagnostic tools to identify molecular groups, copy number alterations, and gene fusions, have been prospectively validated within the BIOMECA-consortium.^80^^,^^95^ Therefore, a solid evidence basis to use these tools in prospective trials has been generated. Whereas the 2 large international trials SIOP EP II and ACNS0831 were not stratified according to molecular groups or biomarkers, the next generation of clinical trials will certainly include molecular markers as a central stratification tool.

## Clinical Trials in Ependymoma, the Uncertain Role of Chemotherapy and New Strategies for Local Tumor Control

In the late 20th century, the first larger prospective clinical trials for children and adolescents diagnosed with ependymoma were initiated. These clinical trials were not ependymoma-specific but included all kinds of *primitive neuroectodermal tumors* “PNETs” and proposed the same treatment strategies independent of the histopathological diagnosis.^96–98^ To our knowledge, the first systematic trial for children suffering from ependymoma was CCG-942, which compared the use of maintenance chemotherapy after resection and radiation therapy in medulloblastoma and ependymoma patients.^96^ This trial recruited from 1975 onwards and although it failed to demonstrate a benefit of a chemotherapeutic combination of CCNU, Vincristine, and Prednisone for ependymoma patients, it was an important milestone for the field overall. Important achievements of this and the following umbrella trials were to enable structured registration of all affected patients, to establish standard diagnostic procedures and to evaluate standardized treatment regimens including maximal safe surgery, chemotherapy, and irradiation especially regarding their efficacy and toxicity. These early clinical trials observed that the objective response to chemotherapy was limited for ependymoma compared to medulloblastoma or other PNETs.^13^^,^^96^^,^^99^^,^^100^ Quickly, the importance of both complete macroscopic and radiological tumor resection, as well as the early application of irradiation, was underlined by several trials.^97–108^ For instance, CCG-9942 reported that some patients could benefit from chemotherapy and showed that patients with subtotal resection suffered from dismal outcomes, therefore recommending second-look surgery in these cases.^109^ Therefore, in the 1990s, subsequent umbrella trials started to propose distinct treatment protocols for ependymoma. Radiation therapy was introduced to complement neurosurgery already in the beginning of the 20th century for pediatric brain tumors.^110^ The technique and strategy developed at that time was the irradiation of the entire neuroaxis covering the whole brain and the full spinal cord.^111^ Even if those treatments were originally employed for medulloblastomas, procedures were adapted to all other types of pediatric brain tumors in the first treatment protocols for brain tumors. However, in the pilot study HIT-SKK87, the rate of spinal seedings seen in ependymoma was low.^98^^,^^112^ In the ‘90s, for instance, within the HIT-SKK92 protocol, all infratentorial ependymoma still received neuroaxis irradiation, whereas supratentorial ependymoma was treated with local tumor irradiation only. After the incidence of CNS dissemination in several international trials was again low, radiation therapy for infratentorial localized ependymoma was restricted to local tumor bed irradiation in the HIT 2000 protocol.^113^ Another trial that showed the prognostic significance of adjuvant radiotherapy was the second AIEOP-trial, which investigated the impact of a radiation boost in patients with a residual tumor.^114^ Nowadays, local highly conformal radiation is the standard strategy for all localized ependymoma patients if adjuvant local therapy is needed. Notably, the introduction of proton therapy showed significantly less long-term sequelae while being equally effective to traditional photon therapy.^115^^,^^116^ With regard to systemic therapy, intensive induction chemotherapy was used in the 1990s for young patients under the age of 3 to 6 years to postpone irradiation. Nevertheless, with increasing knowledge on safety and toxicity of irradiation combined with the awareness of its efficacy in ependymoma, the age limit for radiotherapy was subsequently reduced to 12 months in current protocols.^108^^,^^114^^,^^117–119^ Merchant et al. also showed that a margin of 10 mm for local radiation was safe in non-metastatic ependymoma.^108^ ACNS0121 showed that the EFS for children younger than 3 years of age can be significantly improved by immediate adjuvant radiotherapy.^119^ Interestingly, the same trial reported favorable OS for some patients with completely resected supratentorial ependymoma, classic histology and without adjuvant therapy, suggesting that a small group of patients may be curable with surgery alone. Several studies investigated whether a subset of patients could be treated with postoperative chemotherapy alone to avoid or delay radiotherapy, most importantly POG-8633, CCG-9921, CNS9204, HIT-SKK87/92, and a trial by the SFOP.^98^^,^^101^^,^^104^^,^^120^ Although these trials showed favorable outcomes for a subset of patients without radiation, the findings were not translated into standard daily practice due to the overall modest effect of chemotherapy in other studies. For instance, the recently published results from the E-HIT2000 trials did not find a survival advantage for postoperative chemotherapy in either patients after complete resection or with residual tumor.^92^ The role of chemotherapy apart from bridging to irradiation for very young patients and bridging to re-surgery for patients with significant residual tumor remains unclear and is subject to investigation even in current clinical trials, particularly because a relevant proportion of patients cannot be cured by irradiation alone.^92^^,^^95^^,^^109^^,^^121–123^ Clinical practice shows that in selected cases, chemotherapy can improve the operability of ependymoma.^119^ However, the role of neo-adjuvant chemotherapy in cases with extensive tumor manifestation in between surgical interventions remains unclear. In summary, a statement by Eric Bouffet from 1998 remains largely true to date: “The role of chemotherapy in ependymoma remains unknown.”^99^

**Figure 4.**
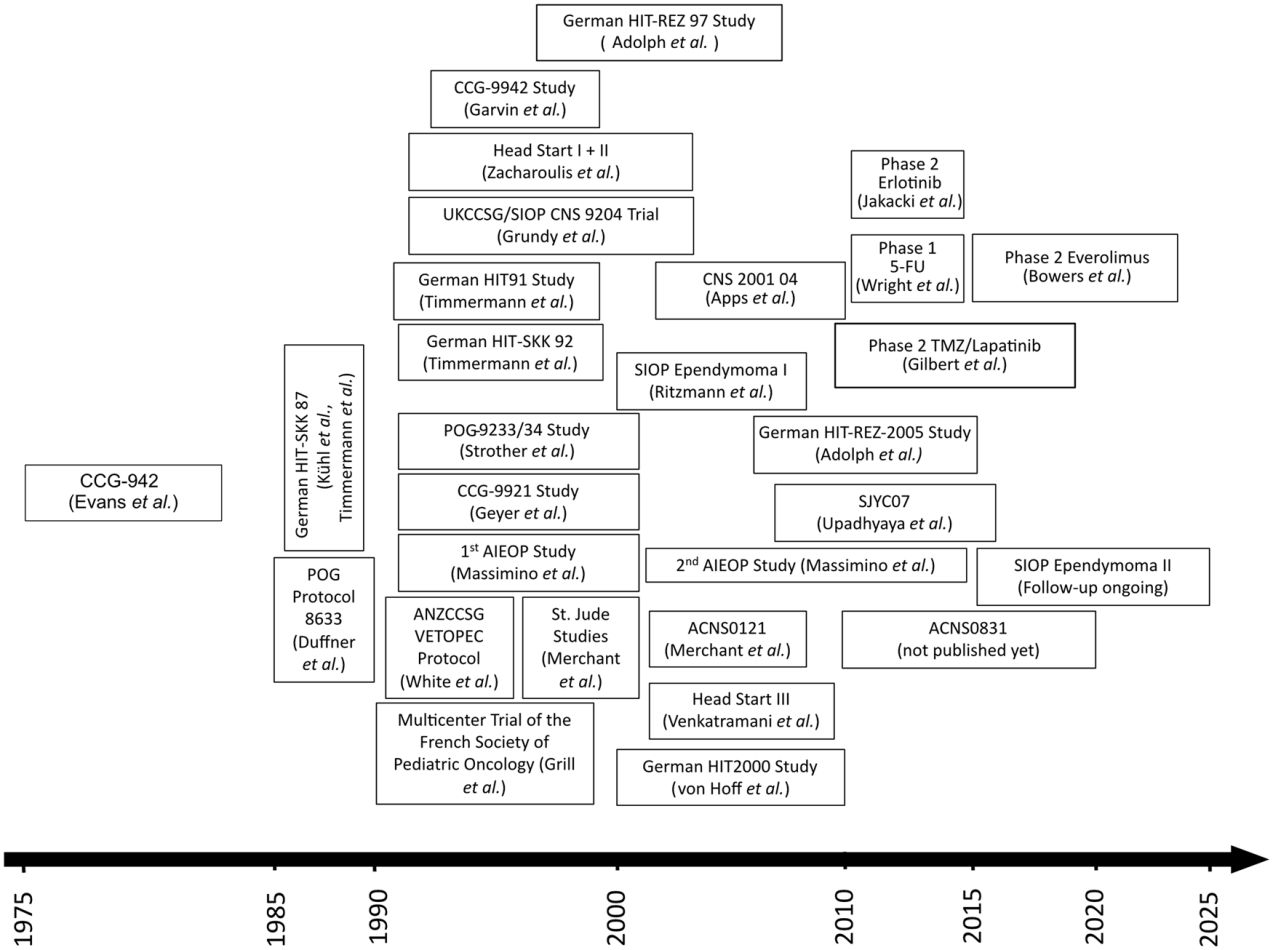
An overview of clinical trials that have shaped the current treatment strategies for ependymoma.

After 5-fluorouracil (5-FU) showed promising results in preclinical studies, a phase 1 trial showed favorable pharmacokinetic properties of 5-FU in children with recurrent ependymoma, sparking ongoing interest in developing this drug as a potential new treatment.^124^^,^^125^ A phase 2 trial investigating the use of the mTOR-inhibitor Everolimus showed negative results, representing a rare example of an ependymoma-specific trial using a targeted therapy.^126^ Importantly, in 2021 the first prospective phase 2 trial in adult ependymoma patients reported objective responses in 16% of patients accompanied by improved quality of life in most patients for the use of oral temozolomide and lapatinib.^127^ The latest trials initiated during the last decade again aimed at specifying the impact of certain chemotherapy regimens in ependymoma and enabling comprehensive accompanying biological studies (Figure 4). Still, the scientific community is awaiting the first prospective clinical trial for ependymoma based on molecular stratification. Therefore, to date, all conclusions on the efficacy of certain treatment strategies in the context of the molecular groups, subgroups, and types are based on retrospective analyses only.^95^^,^^114^ Furthermore, new clinical trials for ependymoma evaluating targeted therapies are widely lacking.

Given the fact that frequently neither systemic therapy nor irradiation is able to prevent progression of radiologically visible residual tumor or local recurrence after total resection, the role of complete macroscopic resection with ideally microscopically tumor-free resection margins is extremely important. Since surgical performance is highly individualized due to different inter-individual surgical skills and in addition dependent on the amount of surgical experience with the specific peculiarities of ependymoma, it remains a matter of concern that the treatment component with the highest efficacy has the naturally lowest level of standardization across a country or a continent. In order to address this issue, the SIOP-EP II trial made a national pediatric neurosurgical reference panel opinion mandatory for all patients with visible residual tumor prior to radiation therapy and in the context of recurrence and potential re-irradiation. Critically, those expert opinions were intended to support decision-making for the local neuro-oncology teams or to prompt the transfer of the respective patients to national pediatric neurosurgery reference centers, having higher caseloads, more experience, and possibly better technical expertise and equipment. In the future, pediatric neurosurgical reference centers may play a role of growing importance in keeping the delicate balance between radical surgery on one side and the maintenance of a sufficiently high quality of life and neurological functionality on the other side for ependymoma patients. In this context, an analysis of the role of surgical reference panels (eg, within the SIOP EP II trial) on the rate of secondary surgery, the effect of re-surgery on patient morbidity and quality of life, and the length of disease control and survival duration would be particularly interesting.

## Recurrent Ependymoma—An Unsolved Clinical Challenge

Despite all efforts to optimize primary therapy, 40%-50% of ependymoma patients experience disease recurrence.^92^^,^^119^^,^^128^^,^^129^ Whereas relapses frequently arise in the first 2 years after diagnosis, late relapses more than 10 years later have been reported, implicating that follow-up time for ependymoma needs to be sufficiently long.^114^^,^^128^^,^^130^^,^^131^ Outcome for recurrent intracranial ependymoma remains poor, and the overwhelming majority of patients will eventually succumb to their disease.^12^^,^^128^^,^^130^^,^^132–134^ Whereas some recurrent ependymomas will lead to a rapid progress and short OS, frequently a chronic course of disease with multiple relapses and eventually no remaining therapeutic options will occur. Similar to the primary setting, local disease control by re-surgery and re-irradiation may offer prognostic benefit. Patients in whom GTR can be achieved show significantly longer survival across several analyses.^12^^,^^132^^,^^135–137^ Compared with surgery, re-irradiation shows more mixed results but is generally accepted as a therapeutic option and has been reported to result in longer survival in cases with STR, to improve local tumor control and to offer potential benefit in the setting of metastatic spread when applied as CSI.^12^^,^^130^^,^^132^^,^^136^^,^^137^ Although most studies report good tolerability and few severe complications at least for the first attempt of re-surgery and re-irradiation, the risk of severe morbidity in a setting in which curation remains the exception should be carefully discussed with the patient and/or his caregivers. So far, systemic therapy in the recurrent setting showed disappointing results independently of individual therapeutic regimens for both classical chemotherapeutic agents and targeted therapy.^11^^,^^12^^,^^133^^,^^138–140^ Generally, the overall evidence base for the recurrence setting is weak, since the applied therapeutic strategies reported in the literature are very variable, and only few clinical trials, such as the HIT-REZ 97 and 2005, tested predefined treatments in a ­prospective manner.^11–13^^,^^141^

## The Role of Patient Organizations in Moving Ependymoma Research Forward

Similar to other rare cancer entities, particularly in the field of (pediatric) neuro-oncology, ependymoma research has traditionally been challenging to conduct due to a lack of funding, public interest and coordinated efforts between isolated research groups. Therefore, patient organizations and advocates were crucial to fuel meaningful progress regarding ependymoma research. The most prominent ependymoma-focused organization serving the community over the past 2 decades is the US American Collaborative Ependymoma Research Network (CERN) Foundation. Established in 2006, the CERN Foundation, which became a program of the National Brain Tumor Society (NBTS) in 2020, is the largest ependymoma-focused organization that offers education, awareness, and outreach activities, and has a dedicated research funding strategy. The concept for the CERN Foundation was envisioned by Dr Mark Gilbert when Dallas Mathile, a patient at MD Anderson Cancer Center, was facing a second recurrence of an anaplastic ependymoma. Notably, CERN led to the formation of a 17-center international clinical trials network, and the creation of an ependymoma-specific biobank. Support from the CERN Foundation has led to over 60 peer-reviewed publications in leading medical journals, amongst them landmark studies like the initial description of the *ZFTA-RELA-*fusion in supratentorial ependymoma, the first prospective ependymoma-trial in adults, a phase 1 study investigating 5-FU in pediatric patients, and the establishment of the molecular classification system for ependymoma.^2^^,^^49^^,^^124^^,^^127^ Other organizations that have contributed to ependymoma-focused research efforts include groups like the Pediatric Brain Tumor Foundation (PBTF), NBTS, The Morgan Adams Foundation, Robert Connor Dawes Foundation, The Lilabean Foundation, Alex’s Lemonade, Ependymoma Research Foundation, Fighting Ependymoma, American Brain Tumor Association, PNOC Foundation, Tommy Strong, the Deutsche Kinderkrebsstiftung, Ein Kiwi gegen Krebs-foundation, Fight Kids Cancer, The Brain Tumour Charity, and many more.

The history of ependymoma research is a great example how disease-specific research funding that is driven by patient organizations is imperative to generate momentum in scientific discovery, to build a base of expert clinicians and scientists and to incentivize them to stay in the field. Furthermore, initial grants by smaller patient organizations frequently open doors for larger grants to build on these initial investments. In addition, advocacy groups focused on tumor specific support serve as a connection to the patient community, bringing awareness to clinical trials and other research opportunities they might participate in.

## Outlook: 160 Years and Counting—Overcoming Challenges to Cure Ependymoma

The last decade has seen unprecedented progress with regard to our understanding of the biological foundations of ependymoma. As presented in this review, these new insights have been successfully translated into clinically diagnostics, prognostication, and risk stratification. However, treatment options for ependymoma have not evolved in parallel and remain in principle the same as they were before the advent of molecular diagnostics. Whereas the unresolved question regarding the benefit of chemotherapy in intracranial ependymoma may be answered in the near future by the currently unpublished SIOP EP II and ACNS0831 trials,^142^^,^^143^ completely new therapeutic strategies are direly needed. As shown by the results of the recently published E-HIT 2000 trial, further conventional therapy intensification does not improve outcome in high-risk patients.^92^ Overall, it seems highly unlikely that patients with dismal risk constellations, such as PF-A with Chr. 1q-gain or Chr. 6q-loss, will be curable with conventional treatment options. This is especially true for patients suffering from relapse, who represent a collective for whom effective salvage strategies are sparse. On the other hand, there may exist more favorable risk constellations for whom current treatment strategies could be de-escalated. This is especially true for radiotherapy, which despite significant technological progress still frequently results in life-long sequelae.

The molecular classification of ependymoma represents an asset for designing the next generation of ependymoma trials. However, it also raises new challenges that need to be addressed. First of all, every stratification system that accounts for molecular heterogeneity will result in smaller trial strata, therefore requiring larger patient collectives and/or longer recruiting phases to arrive at statistically meaningful conclusions. In addition, the optimal combination of risk markers is currently unclear. For instance, copy number aberrations, like Chr. 1q-gain or Chr. 6q-loss, only partly overlap with methylation subtypes in PF-A. Furthermore, the question on how to combine clinical risk factors, like incomplete resection status, with biological risk factors, requires further discussion. A clinical meta-analysis that combines different sets of prospectively collected high-quality trial data from the European and Northern American study groups could be a major step forward in arriving at a definitive answer to these important questions. Without any doubt, molecular diagnostics will represent a cornerstone for the stratification system of the next generation of ependymoma trials.

Histopathological grading of ependymoma remains a controversial topic and has done so for a long time.^99^ Several studies did not show any correlation between tumor grade and prognosis or presented conflicting results, with grading affecting either event free or overall survival.^45^^,^^95^^,^^120^^,^^144^^,^^145^ This included the so far most comprehensive investigation on grading in ependymoma, which incorporated a step-wise process of reanalyzing the cases of 4 European trials by several experienced neuropathologists.^45^ Recently published data from the German HIT-network suggests that histopathologic grading may not be independent from molecular grouping.^92^ However, other studies, amongst them ACNS0121 and most recently ACNS0831, reported prognostic significance of grading in ependymoma, and especially in adult populations, grading is still widely used in daily clinical routine.^10^^,^^105^^,^^118^^,^^119^^,^^129^^,^^146–148^ Varying levels of experience and differing case numbers between individual neuropathologists may partly explain these conflicting results. To date, molecular diagnostics offer novel, technically robust, and observer-independent tools for risk stratification, and it remains to be seen whether grading will show additional value to future stratification systems.

As pointed out, achieving local tumor control is of utmost importance when treating ependymoma. Therefore, the introduction of pediatric neurosurgical reference centers and reference panels may represent a comparatively easy way to potentially improve the outcomes and quality of life for ependymoma patients. Even the most specialized neurosurgical centers will likely not overcome the inherent biological aggressiveness of certain groups and subtypes of ependymoma. However, they represent a natural option to standardize or at least homogenize the initial neurosurgical treatment efficacy and to optimize the chances for long term disease control by adjuvant treatments that follow surgery.

Unfortunately, early-phase clinical trials that included ependymoma in the past have mostly been “mixed bag”—studies that enrolled ependymoma alongside multiple other malignancies, therefore making it difficult to derive meaningful conclusions on drug efficacy. Biologically informed, ependymoma-only phase 1/2 studies or “window of opportunity”—designs as part of larger trials will be necessary to move the most promising early phase drug candidates forward. However, these are currently rare in ependymoma. Although multiple preclinical studies have identified potential vulnerabilities, ranging from gene fusions and super-enhancers to epigenetic and metabolic dysregulation, most of these targets, like *ZFTA-*fusions or *EZHIP* in PF-A, are currently non-druggable.^8^^,^^63^^,^^149–153^ As patient-derived in vitro and in vivo models of ependymoma are notoriously hard to cultivate, especially from primary tissue, there is also a relative lack of preclinical models. Other therapeutic modalities, such as immunotherapy, radiosensitizers, or antibody-drug-conjugates, may become relevant in the future. Two ependymoma patients treated with HER2-specific Chimeric Antigen Receptor T (CAR T) cells showed a good tolerance profile and evidence of local immune activation, pointing toward cellular immunotherapy as a potential future road for ependymoma treatment.^154^ In addition, a recent study suggested GD2 as a new potential target for CAR T-cell therapy.^155^ The establishment of immunotherapy in ependymoma may be facilitated by a growing understanding of the tumor microenvironment and intratumoral heterogeneity, which is increasingly investigated using the latest generation of “omics”—technologies, such as single-cell sequencing and spatial transcriptomics.^87^^,^^156–161^

A particular challenge in conducting clinical trials in ependymoma lies in the fact that several molecular groups occur in both adults and children, most importantly PF-B and SP-MYCN, but also SP-MPE and ST-ZFTA.^10^^,^^162–164^ Overall, around 1 in 4 adult patients with ependymoma will suffer from relapse.^10^ For these patient collectives, shared clinical trials between adult and pediatric neuro-oncology groups will be necessary. Whereas SP-MYCN has early been identified as a high-risk entity, PF-B has long been understood to harbor a comparably good prognosis. However, recent evidence suggests that also in PF-B, late relapses can occur, resulting in significant morbidity and mortality.^165^ Apart from SP-MYCN, spinal ependymoma mostly occurs in older adults and represents a particularly understudied disease group. To our knowledge, no larger drug trial has ever been conducted specifically in spinal ependymoma, and treatment recommendations are mostly based on anecdotal evidence. To date, no promising drug candidates have been reported for any group of spinal ependymoma or for PF-B. Therefore, it seems likely that the next step for these diseases may be the establishment of preclinical models as well as clinical registries that prospectively collect treatment data to develop an evidence-based therapeutic backbone, and to define entity-specific risk stratification models similar to what has been achieved for pediatric intracranial ependymoma.

Ependymoma research has come a long way since its first steps in the 19th and 20th centuries. Whereas the current molecular classification system has revolutionized the way we diagnose and stratify ependymoma, the development of new and risk-adapted therapeutic options has lagged behind. There are many reasons for this ongoing shortcoming, and some of these have been mentioned in this review: the lack of preclinical models and promising drug targets particularly in relapses and high-risk patients, the heterogeneity of surgical expertise and difficulty of standardising surgical interventions across clinical trials, the uncertainty on the role of conventional chemotherapy, and the necessity to conduct age-spanning clinical trials in some of ependymoma subgroups. In order to arrive at the ultimate goal of finding cures while simultaneously reducing morbidity for all types of ependymal tumors, we must overcome these challenges, which will only be possible through active patient involvement as well as extensive international, multi-institutional, and interdisciplinary cooperation.

## Data Availability

No new data were generated or analyzed in support of this research.
